# Molecular characterization and *in silico* evaluation of surfactins produced by endophytic bacteria from *Phanera splendens*

**DOI:** 10.3389/fchem.2023.1240704

**Published:** 2023-08-07

**Authors:** Eleane Monaliza de Cerqueira de Souza, Maycon Vinicius Damasceno de Oliveira, José Edson de Sousa Siqueira, Daniela Cristiane da Cruz Rocha, Anderson do Nonato Rosario Marinho, Andrey Moacir do Rosario Marinho, Patrícia Santana Barbosa Marinho, Anderson H. Lima

**Affiliations:** ^1^ Laboratório de Bioensaios e Química de Microrganismos, Instituto de Ciências Exatas e Naturais, Universidade Federal do Pará, Belém, Pará, Brazil; ^2^ Laboratório de Planejamento e Desenvolvimento de Fármacos, Instituto de Ciências Exatas e Naturais, Universidade Federal do Pará, Belém, Pará, Brazil; ^3^ Laboratório de Enteroinfecções Bacterianas, Instituto Evandro Chagas, Ananindeua, Pará, Brazil

**Keywords:** surfactins, *Pf*Sir2A, *Plasmodium falciparum*, *Phanera splendens*, molecular dynamcis

## Abstract

The *Phanera splendens* (Kunth) Vaz. is a medicinal plant that is used in traditional medicine for the treatment of various diseases, such as malaria. This plant presents highly efficient endophytic bacterial isolates with biocontrol properties. *Bacillus* sp. is responsible for the production of a variety of non-ribosomal synthesized cyclic lipopeptides which highlight the surfactins. Surfactins have a wide range of antimicrobial activity, including antiplasmodial activity. There is scientific evidence that surfactin structure 2d-01 can be a potent inhibitor against *a Plasmodium falciparum sirtuin (Sir2)* by acting on the Sir2A protein as the target. The *Pf* genome encodes two known sirtuins, *Pf*Sir2A and *Pf*Sir2B, where *Pf*Sir2A is a regulator of asexual growth and var gene expression. Herein, we have identified six surfactins produced by endophytic bacteria and performed *in silico* analysis to elucidate the binding mode of surfactins at the active site of the *Pf*Sir2A enzyme. Among the characterized surfactins, 1d-02 showed the highest affinity for the *Pf*Sir2A enzyme, with binding energy values equal to −45.08 ± 6.0 and −11.95 ± 0.8 kcal/mol, using MM/GBSA and SIE methods, respectively. We hope that the information about the surfactin structures obtained in this work, as well as the potential binding affinity with an important enzyme from *P. falciparum*, could contribute to the design of new compounds with antimalarial activity.

## Graphical Abstract

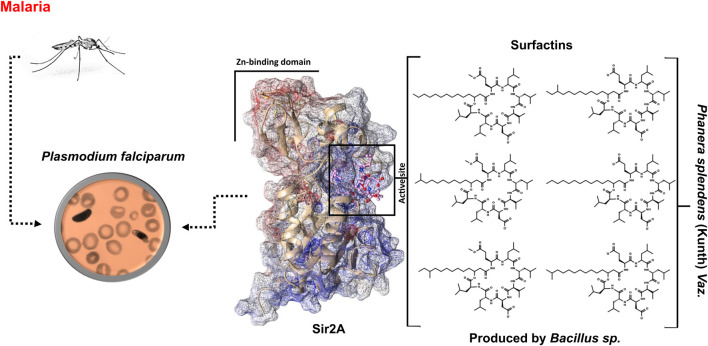

## Introduction

The search for molecules that can be used to enrich the therapeutic arsenal against the most diverse diseases goes through several stages. Traditional medicine is one of these stages since the use of traditional knowledge is a useful guide in the search for bioactive compounds (Tran et al., 2020). The *Phanera splendens* (Leguminosae) is a valuable medicinal plant native to Brazil used in traditional medicine for various diseases treatment such as liver and kidney infections, diarrhea, body pain, gastric disorders, diabetes, and malaria (Filho, 2009; Benjamim et al., 2020; Pina et al., 2021). The presence of endophytic microorganisms within the plant is known to give rise to bioactive compounds akin to those found in their host (Gouda et al., 2016). This approach is in line with the rational use of natural sources, as it makes it possible to obtain bioactive compounds without the need to cut a single tree (Ramos et al., 2022).

Particularly, endophytic microorganisms of the *Bacillus* species have been identified as producers of secondary metabolites, specifically lipopeptides, which exhibit broad bioactivity (Mondol et al., 2013; Kaspar et al., 2019). These cyclic lipopeptides are composed of a β-amino or β-hydroxy fatty acid integrated into a peptide moiety, with variations in the amino acid sequence and fatty acid chain branching, leading to three distinct families: iturin, fengycin, and surfactins (Kim et al., 2017; Barale et al., 2022; Tank and Pandya, 2022). Among these, surfactins stand out due to their diverse applications, acting as antibiotics, antivirals, anticancer agents, antifungals, and even possessing anti-inflammatory properties (Kracht et al., 1999; Das et al., 2008; Wu et al., 2017; Zhao et al., 2017; Meena et al., 2020a; Meena et al., 2020b; Vicente et al., 2021). They are regarded as potent biosurfactants, making them a subject of significant interest (Vicente et al., 2021; Nagtode et al., 2023).

In the context of Antimalarial agents, surfactins have shown particular promise. They have been reported as potent inhibitors of the intraerythrocytic growth of *Plasmodium falciparum*, the parasite responsible for severe malaria, with a 50% inhibitory concentration of 35.2 µM. The key target of surfactins in the parasite is a NAD-dependent deacetylase Sir2 (*Pf*Sir2A), which plays a regulatory role in asexual growth and var gene expression (Chakrabarty et al., 2008; Zheng, 2013). The *P. falciparum* genome encodes two known sirtuins, *Pf*Sir2A and *Pf*Sir2B, but it is *Pf*Sir2A that has a crucial role in the development of malaria (Frye, 2000; Kumar et al., 2020; Sato, 2021). Malaria’s clinical manifestations are associated with the parasite’s robust proliferation within the mammalian host’s bloodstream, underscoring the importance of finding effective antimalarial agents that can target *Pf*Sir2A (Kumar et al., 2020).

In our study, we focused on the endophytic bacteria *Bacillus sp*. associated with the *P. splendens* plant, identifying six surfactins produced by these bacteria. Our investigation revealed how surfactins bind to the active site of the *Pf*Sir2A enzyme, providing a potential mechanism for their antimalarial activity. To gain insights into the binding interactions between surfactins and the *Pf*Sir2A enzyme, we employed computational methods to analyze their structures and energetics. This information helped to elucidate the binding mode of surfactins and lead to the potential development of new antimalarial agents that target the *Pf*Sir2A enzyme.

## Materials and methods

### Isolation and identification of endophytic bacteria B.1.5C1 and B1.11F (1.2)

*P. splendens* leaves and stems were collected from the Embrapa Amazônia Oriental (Pina et al., 2021). The surface sterilization was conducted according to the procedure from Romeiro (2001) with some modifications. The samples were washed immediately with tap water to remove the attached dust and soil and then sterilized successively with hexane (Tedia Brazil^®^) for 1 min, 70% ethanol for 30 s, and 2% sodium hypochlorite for 4 min. After being rinsed three times with sterilized distilled water, the outer tissues of the samples were removed with a sterilized scalpel blade, and the remainders were cut into 0.5-cm-long fragments. The rinsed fragments were vertically halved and incubated on BHI broth (HiMedia, Mumbai, India): Heart Brain Infusion (prepared according to the manufacturer). After turbidity of the broth, an aliquot was plated on MacConkey Agar, SS Agar, and Nutrient Agar for the isolation of bacteria until the colonies appeared around the segments. The isolates were purified by streak culturing, and the distinct colonies were subcultured in an appropriate culture medium. At the same time, isolates were stored at −80°C in cryogenic tubes containing 30% glycerol until used. To evaluate the effectiveness of the above sterile process, a similar procedure without surface sterilization was performed as a negative control and aliquots of 0.1 mL of the last rinsed water were inoculated on BHI medium as a positive control. Morphologically different colonies were subjected to identification by biochemical analysis using the BCL card of the Vitek2 system (bioMérieux, Marcy l'Etoile, France) (Celandroni et al., 2019).

### Preparation of extracts of endophytic bacteria B.1.5C1 and B1.11F (1.2) and obtaining of compounds

It was done based on Ben Abdallah and Khiareddine (2015) (Ben Abdallah and Khiareddine, 2015) and Conti et al. (2016) (Conti et al., 2016) with some adaptations. In brief, pre-cultured bacterial B.1.5C1 and B1.11F (1.2) were inoculated in BHI medium and incubated for 7 days at 32°C in the orbital shaker (100 rpm). After incubation, bacterial biomass was separated by centrifugation at 10,000 g for 15 min. The supernatant was filter sterilized with a 0.22 μm sterile filter unit. The collected supernatant was extracted twice using an equal volume of ethyl acetate in a separating funnel by shaking vigorously for about 5 min. After this, we left to rest for 15–20 min. After 15–20 min, the aqueous and organic phase were separated and the organic phase was collected. The organic phase obtained or collected was subjected to rotary evaporation under vacuum to obtained to crude organic extracts of 0.0484 g for *Bacillus* sp. B.1.5C1 and 0.454 g for *Bacillus* sp. B1.11F. From the ethyl acetate extracts, after fractionations on Sephadex LH-20 chromatographic column eluted with methanol, **1** (20.4 mg) of B.1.5C1 and **2** (33.8 mg) of B.1.11F (1.2) compounds were obtained.

### NMR and MS analysis

^1^H (400 MHz) and ^13^C (100 MHz) NMR spectra were performed on a Bruker Ascend 400 (Bruker, Fällanden, Switzerland). **1** was solubilized in DMSO-d6 and **2** was solubilized in CDCl_3_ to record NMR spectra. The chemical shifts are given in delta (δ) values and the coupling constants (*J*) in Hertz (Hz), and the solvent signal (DMSO-d6 or CDCl_3_) was used as a reference. Mass spectrum was acquired on an Acquity tandem quadrupole detector (TQD) mass spectrometer (Waters, Milford, MA, United States) equipped with an electrospray ionization (ESI) source operating in positive or negative mode.

### HPLC analysis

High-Performance Liquid Chromatography (HPLC) analysis was performed using an Alliance e2695 system (Waters, Milford, MA, United States) equipped with an autosampler and photodiode array detector (DAD, Waters 2998) and a SunfireTM C18 column, 5 μm (4.6 × 150 mm) (Waters^®^) attached to a guard column (SunfireTM C18, 5 μm, 4.6 × 20 mm, Waters^®^). The mobile phase consisted of a gradient of H_2_O (A):CH_3_CN (B) with a flux rate of 2 mL/min for 60 min (5%–100% B) and a UV detection wavelength of 210–600 nm.

### Preparation of PfSir2A and surfactin structures for *in silico* analysis

**FIGURE 1:**
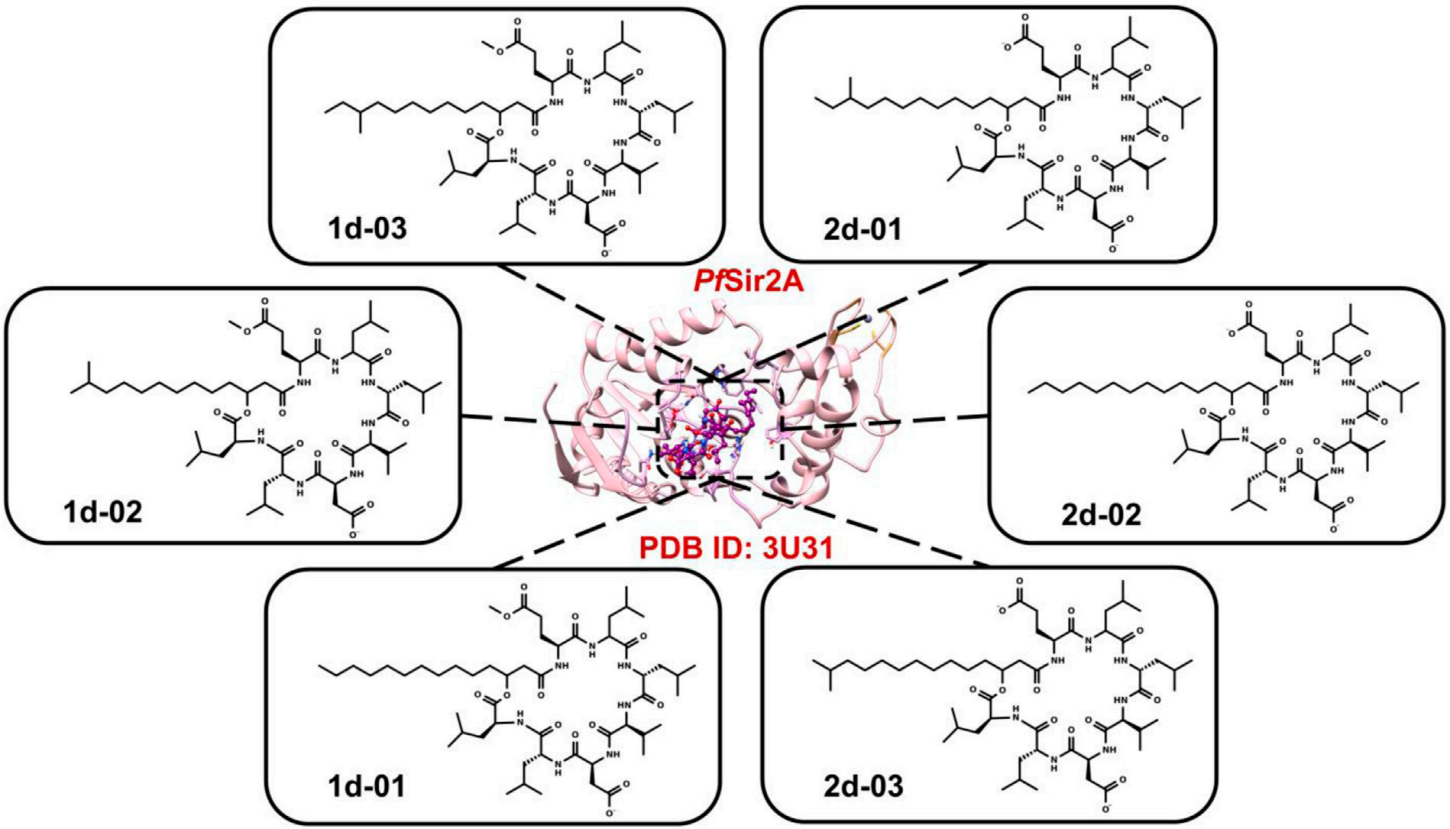
The six 2D structures of surfactins against *Pf*Sir2A (PDB ID: 3U31).

The six surfactin (Table 1) structures (the isoforms of the major compounds) were selected to be analyzed at the active site of *Pf*Sir2A (Figure 1).

**TABLE 1:** The six surfactins ID and their IUPAC name.

Surfactin ID	IUPAC name
1d-01	2-[(3S,6R,9S,12S,15R,21S)-21-(3-methoxy-3-oxopropyl)-3,6,15,18-tetrakis (2-methylpropyl)-2,5,8,11,14,17,20,23-octaoxo-12-(propan-2-yl)-25-undecyl-1-oxa-4,7,10,13,16,19,22-heptaazacyclopentacosan-9-yl]acetate
1d-02	2-[(3S,6R,9S,12S,15R,21S)-21-(3-methoxy-3-oxopropyl)-25-(9-methyldecyl)-3,6,15,18-tetrakis (2-methylpropyl)-2,5,8,11,14,17,20,23-octaoxo-12-(propan-2-yl)-1-oxa-4,7,10,13,16,19,22-heptaazacyclopentacosan-9-yl]acetate
1d-03	2-[(3S,6R,9S,12S,15R,21S)-21-(3-methoxy-3-oxopropyl)-25-(8-methyldecyl)-3,6,15,18-tetrakis (2-methylpropyl)-2,5,8,11,14,17,20,23-octaoxo-12-(propan-2-yl)-1-oxa-4,7,10,13,16,19,22-heptaazacyclopentacosan-9-yl]acetate
2d-01	3-[(3S,6R,9S,12S,15R,21S)-9-(carboxylatomethyl)-3,6,15,18-tetrakis (2-methylpropyl)-25-(9-methylundecyl)-2,5,8,11,14,17,20,23-octaoxo-12-(propan-2-yl)-1-oxa-4,7,10,13,16,19,22-heptaazacyclopentacosan-21-yl]propanoate
2d-02	3-[(3S,6R,9S,12S,15R,21S)-9-(carboxylatomethyl)-25-dodecyl-3,6,15,18-tetrakis (2-methylpropyl)-2,5,8,11,14,17,20,23-octaoxo-12-(propan-2-yl)-1-oxa-4,7,10,13,16,19,22-heptaazacyclopentacosan-21-yl]propanoate
2d-03	3-[(3S,6R,9S,12S,15R,21S)-9-(carboxylatomethyl)-3,6,15,18-tetrakis (2-methylpropyl)-25-(10-methylundecyl)-2,5,8,11,14,17,20,23-octaoxo-12-(propan-2-yl)-1-oxa-4,7,10,13,16,19,22-heptaazacyclopentacosan-21-yl]propanoate

The crystal structure (PDB ID 3U31), deposited by Zhu et al. (2011), was chosen due to having the two substrates, myristoyl peptide H3K9 and NAD, in its active site. These substrates bind to the cleft between the two protein domains. This binding mode of these two substrates is similar to the reported ternary complex structures of other sirtuins (Zhu et al., 2012).

Molegro Virtual Docker (MVD) (version 5.5) was used to perform molecular docking. It uses a heuristic search algorithm, which combines differential evolution with a cavity prediction algorithm (Thomsen and Christensen, 2006). MolDock uses the piecewise linear potential (PLP) as a scoring function, which includes hydrogen bonds and electrostatic terms (Yang and Chen, 2004). Parameters were adjusted by redocking NAD in the *Pf*Sir2A protein (Zhu et al., 2012) (crystal structure accessed under PDB ID 3U31). To validate the crystallographic NAD binding mode, we used a root mean square deviation (RMSD) ≤ 1 Å as a cut-off point (De Oliveira et al., 2020; De Oliveira et al., 2022).

For the docking performance, a grid box with a radius equal to 15 Å was used for the six surfactins, following the location of the active site (X = −6.10 Å, Y = 25.47 Å, and Z = −30.37 Å), with the number of runs equal to 10, the maximum number of interactions equal to 1500; maximum population size equal to 50, and grid resolution equal to 0.30 Å.

### Molecular dynamics (MD) simulation

Conformational dynamics of the systems were performed using the Amber18 package (Case et al., 2018). The general force field (GAFF) was used to treat the ligand structures and the Amber ff14SB to treat the protein structure (Wang et al., 2004; Case et al., 2005). To calculate the atomic charges of the surfactins, we used the Restrained Electrostatic Potential (RESP) protocol adding the Hartree-Fock method with the 6-31G* base set. The *Pf*Sir2A protein contains a small Zn^2+^ binding domain. The divalent cation Zn^2+^ was treated with the Nonbonded Model 12-6-4 Lennard-Jones Type method (Li et al., 2015).

Protonation states of the ionizable residues of the *Pf*Sir2A structure were obtained by calculating pKa at neutral pH using the free online server H^++^ (Anandakrishnan et al., 2012). Cl^−^ ions were added to maintain the electroneutrality of the systems. All systems were solvated in the tLeap module using TIP3P water molecules (Jorgensen et al., 1983; Price and Brooks, 2004). The minimization of the systems was carried out in 4 steps. Firstly, only the counter-ions and water molecules were minimized. Then, protein hydrogen atoms, and after that, protein and ligand hydrogen atoms and the water molecules. Finally, a general minimization of the system without restriction.

We started heating the system from 0 to 300 K by running 200 ps of MD with restrictions on the position of protein-ligand atoms in a constant volume. Before carrying out the production step, all protein-ligand systems were balanced with 300 ps of MD without position restrictions at constant pressure. The temperature was maintained at 300 K by coupling to a Langevin thermostat using a collision frequency of 2 ps^-1^ and the isotropic constant pressure was maintained at 1 bar using the Berendsen barostat. A cutoff of 10 Å was employed for unbound interactions, and the Ewald particle mesh method (PME) was used to calculate long-range electrostatic interactions.

Finally, the MD simulation was performed with a simulation time equal to 500 ns at 300 K. The Root Mean Square Deviation (RMSD) was used to analyze the stability of the complex as a function of simulation time. Besides, the Root Mean Square Fluctuation (RMSF) was used to analyze the mobility of amino acid residues during the last 100 ns of simulation time.

### Binding free energy calculations

To perform the binding free energy calculations between surfactins and the protein, we used the MM/GBSA method (Genheden and Ryde, 2015) available in the Amber18 package and the solvated interaction energy method (SIE) method available in the SIETRAJ 2.0 program (Sulea and Purisima, 2012). The last 100 ns (10,000 frames) of the trajectories of the MD simulations were used to calculate binding free energy and binding free energy decomposition.

## Results and discussion

### Identification of bacteria B.1.5C1 and B1.11F (1.2)

The comparison of spectral data **1** with data from the literature indicates that these are lipopeptides of the surfactin mixtures type (Tanaka et al., 2015; Nasfi et al., 2018). The results obtained in ^1^H NMR spectrum confirm the presence of a long aliphatic chain (CH_2_ in δ_H_ 1.20 and 1.54) and a structure containing amino acids by the N-H signals in δ_H_ 7.20–8.70 and C-H in δ_H_ 4.03–4.50. The chemical shift at δ_H_ 5.03 indicates the presence of an ester group, which can be part of the lactone ring. The singlet at δ_H_ 3.50 is similar to those found in the monoester lipopeptide spectra and suggests the existence of a methoxy group (OCH_3_) at the amino acid residues glutamic acid (Glu) or aspartic acid (Asp) (De Faria et al., 2011; Tanaka et al., 2014). In the ^13^C NMR spectrum, the signals between δ_C_ 11.0 to δ_C_ 20.0 can be observed indicating that the lipid chain of the surfactin, which can present in three different isoforms: linear, normal (*n*-); branched, *iso* and/or *anteiso*. The presence of the *iso*, *anteiso,* and *n*-normal mixture is confirmed by the chemical shifts of the signals referring to the methyl groups at δ_C_ 11.2; 14.0; 18.0, and 19.1, respectively. Exceptionally, the resonance signal at δ_C_ 48.7 indicates that the methoxy group (OCH_3_) is located at the amino acid residue Glu or Asp, confirmed by the signals of the carbonyl carbons between δ_C_ 170.0–174.0, characteristic of amino acid, this being confirmed by the daughter ions of the mass spectrum (De Faria et al., 2011).

**FIGURE 2:**
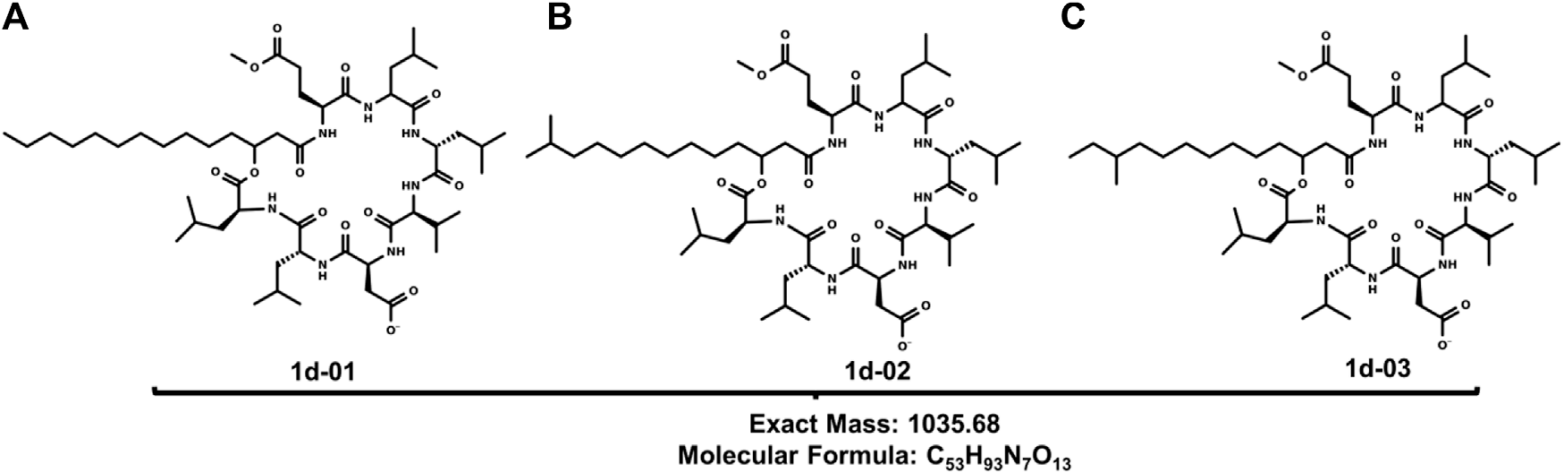
Structures of **(A)** 1d-01, **(B)** 1d-02 and **(C)** 1d-03: Leu6-L-Leu7 C14 [M-H]^+^ produced by B1.5C1.

The positive and negative mode ESI mass spectra for the surfactin derivatives group **1** (a-e) confirm the presence of five surfactin homologs, C11 (C_50_H_87_N_7_O_13_), C12 (C_51_H_89_N_7_O_13_), C13 (C_52_H_91_N_7_O_13_), C14 (C_53_H_93_N_7_O_13_) and C17 (C_56_H_99_N_7_O_13_) with the ion *m/z* 1035 predominant, related to surfactin of aliphatic carbonic chain C14 (Figure 2). These surfactins structures are homologous to each other, differing only by the number of methylene groups (CH_2_–14 Da) in the aliphatic chain (Table 2). These molecules, surfactin isoforms, are found in the genus *Bacillus* (De Faria et al., 2011; Ben Ayed et al., 2014).

**TABLE 2:** Homologs of monomethylester surfactins produced by B1.5C1 analyzed by mass spectrometry.

Surfactin derivatives	Peak (*m/z*) positive mode	Molecular formula	Peak (*m/z*) negative mode	Molecular formula	Fatty acid chain	Amino acid sequence
1a			993.24 [M-H]^-^	C_50_H_87_N_7_O_13_	C_11_	Glu-*O*-Me-Leu-Leu-Val-Asp-Leu-Leu
1b			1006.28 [M-H]^-^	C_51_H_89_N_7_O_13_	C_12_	Glu-*O*-Me-Leu-Leu-Val-Asp-Leu-Leu
1c	1023.54 [M+2H]^+^	C_52_H_91_N_7_O_13_	1022.39 [M-H]^-^	C_52_H_91_N_7_O_13_	C_13_	Glu-*O*-Me-Leu-Leu-Val-Asp-Leu-Leu

1d	1060.52 [M + H + Na]^+^	C_53_H_93_N_7_O_13_	1034.66 [M-H]^-^	C_53_H_93_N_7_O_13_	C_14_	Glu-*O*-Me-Leu-Leu-Val-Asp-Leu-Leu
1e	1078.02 [M + H]^+^	C_56_H_99_N_7_O_13_			C_17_	Glu-*O*-Me-Leu-Leu-Val-Asp-Leu-Leu

The MS/MS spectrum of the most abundant homolog was fragmented. Fragmentation of the ion *m/z* 1037,8 [M + 2H]^+^ resulted in the ions: *m/z* 826,0; 685,8; 678,3; 637,6; 578,1, and 328,3. Surfactin isoforms A and B vary only in the amino acid positions of the peptide chain, Leu and Val, respectively. These differences can be resolved by analyzing the fragment ions.

The most abundant ion (*m/z* 826) corresponds to the mass [M + Me + H -Leu-Leu]^+^ that is verified in the group of the peptide chain that undergoes cleavage in Leu between the carbon-nitrogen bond, leaving the main chain with *N*-terminal of one of the Leu, thus being able to verify the presence of a part of the structure in which one is Leu-Leu suggesting the *isoform* A structure.

In addition, two important *m/z* ions are observed, similar to the studies carried out by Moro et al. (2018) which are *m/z* 578,1 [M - Leu-Leu-Asp-Val - H_2_O] and 678,3 [M - Leu-Leu-Asp - H_2_O]^+^, these fragment ions also show that the methoxy group (O-CH_3_) is found on the amino acid residue Glu. Other important fragments noted to prove the structure are found in ions *m/z* 328,3 [Leu-Asp-Val]^+^, *m/z* 685,8 [M + H–FA - Glu]^+,^ and *m/z* 637,6 [M + H–FA - Glu - H_2_O]^+^ according to Moro and co-workers are characteristic fragments of monomethylester surfactin A.

The surfactin derivatives **2** appeared as a yellow solid and when subjected to HPLC analysis only one UV absorption band was noted at approximately 208 nm. From the comparison with studies carried out with lipopeptides (Tanaka et al., 2015; Moro et al., 2018; Nasfi et al., 2018) and analysis of ^1^H and ^13^C NMR data, it was possible to infer that **2** is constituted by surfactins.

The ^1^H NMR spectrum presented signals between δ_H_ 0.82–0.94 for the terminal methyl groups. The signs between δ_H_ 1.24–1.60 refer to CH_2_ groups and indicate the presence of a long aliphatic chain. The signs in δ_H_ 7.34–8.02 refer to the amide groups (N-H). The α-hydrogen signals of amino acids can be observed between δ_H_ 4.45–5.04.

The ^13^C NMR spectrum shows characteristic signals of carbonyl esters groups between δ_C_ 170.5–175.4 and aliphatic carbons in the δ_C_ 11.3–41.8 region and the analysis also indicates that the lipid tail of the lipopeptide is present as a mixture of isoforms: normal (*n*-), *iso* and *anteiso*, with the different positions of CH_3_ in δ_C_ 11.4; 14.1; 19.1 and 19.3, respectively.

**FIGURE 3:**
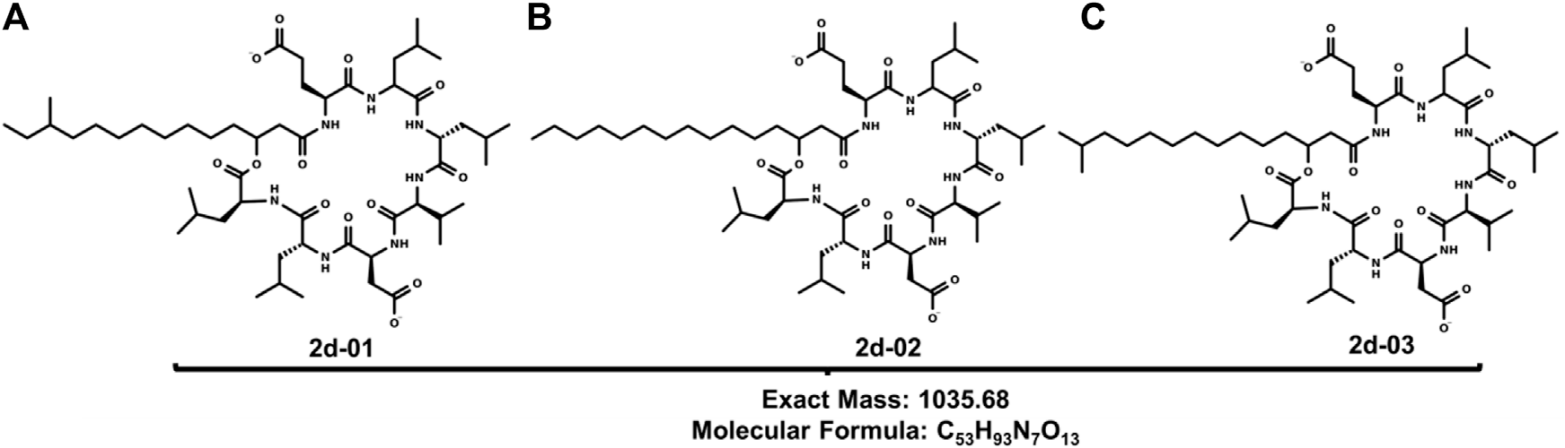
Structures of **(A)** 2d-01, **(B)** 2d-02 and **(C)** 2d-03: Leu6-L-Leu7 C15 [M-H]^+^ produced by B1 11F (1.2).

Similar carbon signals were found in the study by Liu et al. (2014) who also reported a mixture of the *normal*, *iso*, and *anteiso* surfactin forms in *Bacillus subtilis* HSO121 (Liu et al., 2015). The mass spectrum for substances 2a—2f confirms the presence of surfactin isoforms according to literature data (Jacques, 2011; Chen et al., 2015; Liu et al., 2015). Mass spectrum (*full scan*) ESI in negative mode indicates that **2** probably presents a series of surfactins and the molecular structures are shown, respectively, in Table 3. There was a predominance of the ion *m/z* 1034.6 (Figure 3).

**TABLE 3:** Homologs of surfactin produced by B.1.11F (1.2) analyzed by mass spectrometry.

Surfactin derivatives	Peak (*m/z*) [M-H]^+^	Molecular formula	Fatty acid chain	Amino acid sequence
2a	991.56	C_50_H_87_N_7_O_13_	C_12_	Glu-Leu-Leu-Val-Asp-Leu-Leu
2b	1005.62	C_51_H_89_N_7_O_13_	C_13_	Glu-Leu-Leu-Val-Asp-Leu-Leu
2c	1020.00	C_52_H_91_N_7_O_13_	C_14_	Glu-Leu-Leu-Val-Asp-Leu-Leu
2d	1033.97	C_53_H_93_N_7_O_13_	C_15_	Glu-Leu-Leu-Val-Asp-Leu-Leu
2e	1048.33	C_54_H_95_N_7_O_13_	C_16_	Glu-Leu-Leu-Val-Asp-Leu-Leu
2f	1078.64	C_56_H_99_N_7_O_13_	C_18_	Glu-Leu-Leu-Val-Asp-Leu-Leu

In the fragmentation of the ion *m/z* 1034.9 of 2d, cleavage was observed, preferentially, in the lactone ring, producing ions *m/z* 776.8; 692.7; 364.3; 339.0; 306.5; 265.1, and 127.6. These peaks correspond to a loss of amino acid residues in agreement with the surfactin A amino acid sequence.

From the opening of the lactone ring, deprotonation occurs, that is, the loss of hydrogen from the carboxylic group of the side chain of Asp. Then, the transfer of the hydrogen atom from the α carbon of Leu to the oxygen of the ester group occurs through the 1,3 migration of hydrogen. When the carbon bond (from Leu) - oxygen (from the ester group) breaks, the cycle opens. The ion values were identified from the opening of the lactonic ring of the ion of *m/z* 1034.9. The *m/z* 777.8 ion is one of the most important, as it shows the mass of the surfactin A amino acid sequence [Leu-Leu-Val-Asp-Leu-Leu-Glu-H_2_O-H]^-^ and its difference with the molecular ion peak corresponds to 258 Da referring to the mass of the fatty acid side chain of the substance and one less water molecule, proving the initial process of opening the chain. The ions *m/z* 692.7 [M–H–Leu-Leu-Asp]^-^ and 339.0 [M-H-FA-Glu-Leu-Leu-Val]^-^ are characteristic ions of surfactin A fragments. Other important ions are *m/z* 127.6 which exactly represents the molar mass of Glu present in the surfactin structure. The ion *m/z* 306.5 [Leu-Leu-Val-H_2_O-H]^-^ is important in the determination of the structure, as it refers to the tripeptide with the loss of a molecule of water. Another important ion is the most abundant *m/z* 364.3 [M-Leu-Leu-Val-Asp-Leu-Leu]^-^. This fragmentation profile indicates that the structure of the surfactin A lipopeptide is composed of seven amino acid residues Glu-Leu-Leu-Val-Asp-Leu-Leu, with a lactone group linking the terminal Leu amino acid to a −hydroxyl fatty acid chain (Moro et al., 2018).

### Analysis of the active site region and structure of the *Pf*Sir2A

**FIGURE 4:**
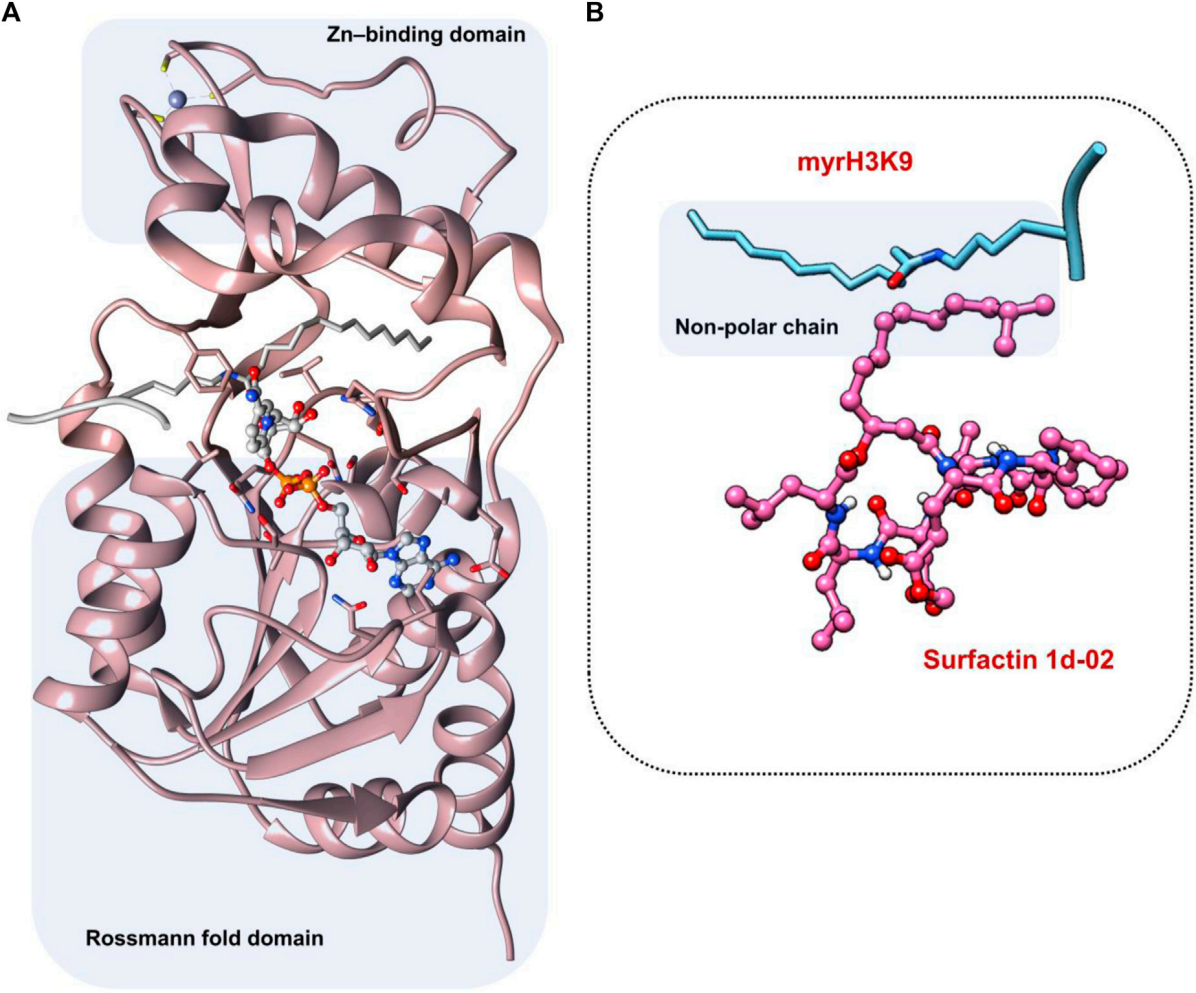
**(A)** Active site region between Rossmann fold domain and Zn-binding domain region **(B)** Similarity between the structure of myrH3K9 and surfactin 1d-02 in the non-polar region.

Sir2A protein has two domains, the Rossmann fold domain, and the Zn-binding domain region, where the active site of the enzyme is in the middle of them (Figure 4A). The protein has a hydrophobic tunnel where the structure of fatty acyl groups is accommodated. On the other hand, the structure of surfactins can be characterized as a ring of linked amino acids and a non-polar carbon-carbon chain. It is important to note that the non-polar chain of surfactins is similar to the N∼6∼-tetradecanoyl-L-lysine (myrH3K9) presented in the *Pf*Sir2A crystal structure (PDB ID 3U31) (Figure 4B).

From docking results, we have observed that the ring region of the surfactin structure was coupled in the NAD region and the non-polar chain was in the hydrophobic tunnel. The MolDock Score values of the poses lie in a range of −227.16 to −173.68 kcal/mol while the NAD redocking score points to −190.06 kcal/mol with an RMSD (from the reference crystal structure) of 0.6 Å (Table 4). Whereas docking results showed similar conformation of all surfactins, we selected only the best surfactin (1d-02) for MD simulations and energetic analysis.

**TABLE 4:** MolDock Score values of surfactins docked against *Pf*Sir2A and the RMSD/MolDock Score values to NAD substrate in the redocking protocol.

Molecular docking	
Surfactin	MolDock score (kcal/mol)
1d-01	−222.16
1d-02	−227.16
1d-03	−206.11
2d-01	−224.07
2d-02	−173.68
2d-03	−176.15
Redocking	

Substrate	MolDock Score (kcal/mol)	RMSD
NAD	−190.06	0.6

### Energetic analysis of surfactin and NAD in *Pf*Sir2A

**FIGURE 5:**
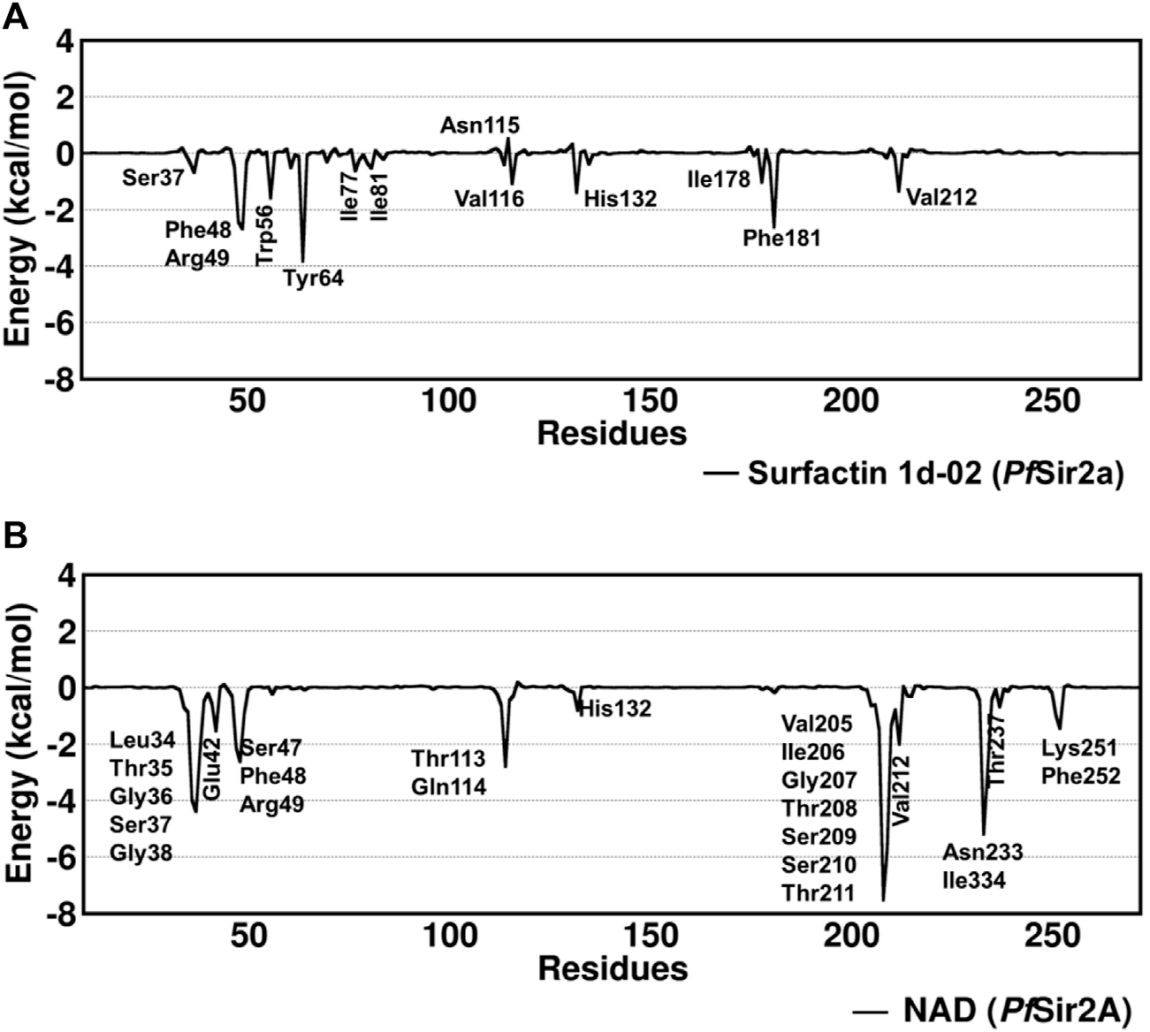
Decomposition of free energy per residue with the MM/GBSA method for **(A)** surfactin 1d-02 and **(B)** NAD considering the main residues with energy decomposition value ≤ or ≥ 0.5 kcal/mol.

The structure of surfactin 2d-02 is already described as a potent inhibitor of intra-erythrocytic growth of *P. falciparum* with 50% inhibitory concentration (IC_50_ = 35.2 µM) (Chakrabarty et al., 2008; Zheng, 2013). The binding free energy analysis showed surfactin 1d-02 with a value of −45.08 kcal/mol employing the MM/GBSA method and −11.95 kcal/mol using the SIE method (Table 5). Per residue decomposition analysis of the binding free energy showed the main amino acid residues that contributed to this value in MM/GBSA analysis. It includes Ser37, Phe48, Arg49, Trp56, Tyr64, Ile77, Ile81, Asn115, Val116, His132, Ile178, Phe181, Val212. Some of them similarly contribute to NAD stabilization, as can be seen in Figure 5.

**TABLE 5:** Binding free energies for surfactin 1d-02 and NAD complex using the MM/GBSA and SIE approach.

Binding free energies			
Method	Energy (kcal/mol)	NAD	Surfactin 1d-02
MM/GBSA	vdW	−75.23 ± 4.1	−72.88 ± 7.4
	EEL	−352.68 ± 15.5	−164.14 ± 15.3
	∆Ggas	−427.92 ± 15.3	−237.02 ± 17.2
	EGB	357.19 ± 14.1	202.05 ± 14.5
	ESURF	−8.26 ± 0.2	−10.11 ± 0.9
	∆G_solv_	348.92 ± 14.1	191.93 ± 14.1
SIE	Inter vdW	−75.24 ± 4.1	−72.88 ± 7.4
	Inter Coulomb	−156.81 ± 6.9	−72.98 ± 6.8
	Reaction Field	146.80 ± 5.6	77.55 ± 5.1
	Cavity	−10.96 ± 0.3	−15.26 ± 1.4
	Constant	−2.89	−2.89
∆G_total_	∆G_total_ (MM/GBSA)	−78.99 ± 6.2	−45.08 ± 6.0
	∆G_total_ (SIE)	−12.97 ± 0.4	−11.95 ± 0.8

**FIGURE 6:**
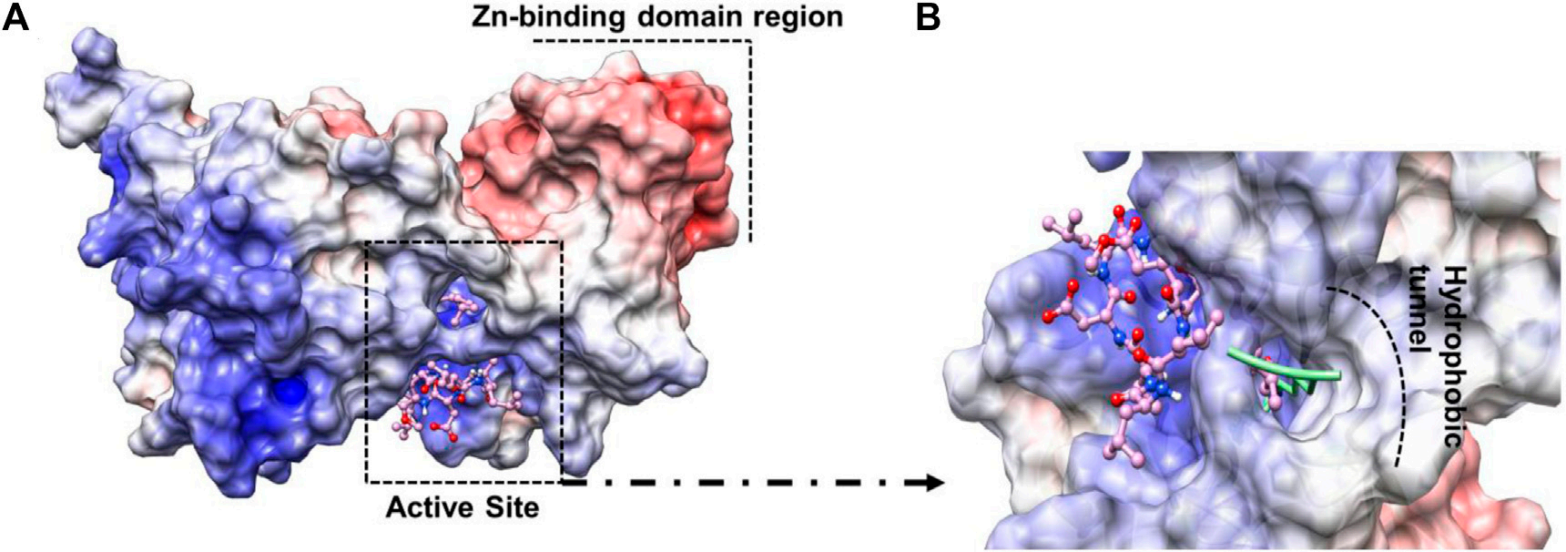
The electrostatic potential maps of **(A)** Sir2A protein complexed with surfactin 1d-02 highlighting the Zn-binding domain region and the active site region between the protein domains and **(B)** surfactin 1d-02 (pink structure) in the active site of protein showing the nonpolar chain in the hydrophobic tunnel and the fatty acyl groups (green structure) accommodated inside that region hydrophobic.

Residues such as Trp56, Ile77, and Val116 are part of the hydrophobic tunnel where the nonpolar chain of surfactin structure is present. Phe48, Tyr64, and Phe181 interact with the carbon atoms that are around the cyclic amino acids in the surfactin structure. From that, we can point out that hydrophobic moieties should be considered in lead optimization once it has a reasonable contribution to the energy value obtained using MM/GBSA. In general, our data demonstrate that the surfactin structure should occupy the NAD active site and the hydrophobic tunnel, where fatty acyl groups are accommodated (Figure 6).

### Molecular dynamics simulation

**FIGURE 7:**
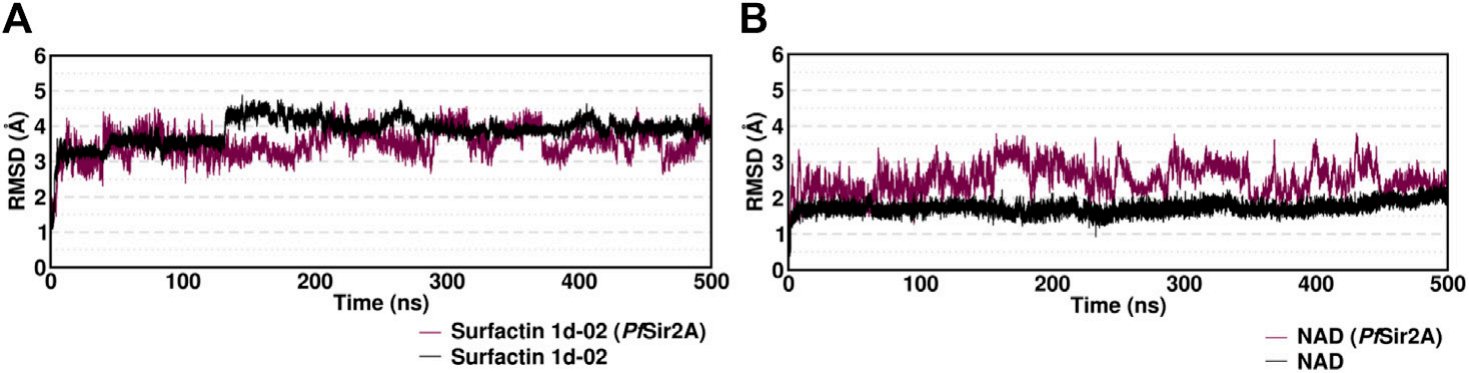
Root mean squared deviation (RMSD) in Angstrom (Å) for the complexes with **(A)** ligand Surfactin/NAD *Pf*Sir2A and **(B)** ligand/substrate NAD of each system over 500 ns of MD simulation time considering only the heavy atoms.

In MD simulations, the conformational changes of the surfactin occurred due to the rotatable bonds present in the structure molecule. RMSD values are within a sensible fluctuation of 1–5 Å showing that the structural equilibrium was accomplished (Figure 7). The surfactin 1d-02 complex showed stability during the simulation with an average value of 3.5 (±0.4) and the NAD complex presented an average value of 2.5 (±0.4) which suggests that the systems remained stable.

**FIGURE 8:**
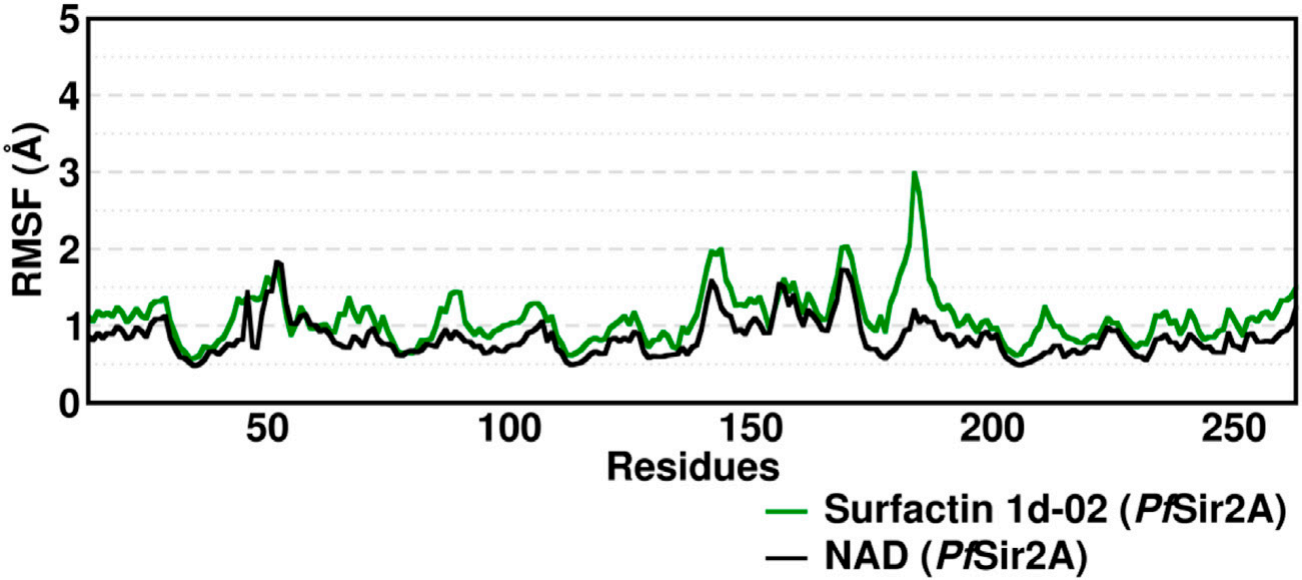
Root Mean Square Fluctuation (RMSF) average of the residue fluctuations obtained along the 400–500 ns MD simulation, using all heavy atoms, for amino acid residues in Surfactins and NAD substrate complexes.

The two domains have loops in protein structure that when analyzed showed small fluctuations between 1 and 3 Å (Figure 8). The active site region presents loops between the domains but they discreetly fluctuate. The movements of fluctuations in this region range up to 3 Å.

In the crystal structure deposited in the PDB (ID: 3U31), the NAD molecule forms hydrogen interactions in the active site region with specific amino acid residues, including Ser37, Glu42, Thr208, Ser209, Asn233, and Phe252, which are conserved in the NAD MD simulations (Supplementary Table S1). Interestingly, during our MD simulations of the six surfactins, we observed that these residues interacted with surfactin with an occupancy of less than 50%. This observation suggests that van der Waals interactions play a crucial role in the surfactins/*Pf*Sir2A complex.

Additionally, we noticed that certain key residues, namely Ser37, Arg49, Gly50, Ser52, Trp56, Tyr64, Val116, Thr208, Ser209, Thr211, and Ser213, were consistently involved in hydrogen interactions with the surfactins during the MD simulations. These interactions occurred in a range of 55%–10% of the simulation frames, with particular emphasis on three main residues: Ser37, Thr208, and Ser209, which also interacted with the NAD substrate (Supplementary Table S1).

These findings indicate that the interactions between surfactins and the *Pf*Sir2A enzyme involve a delicate balance between hydrogen bonds and van der Waals forces. The reduced occupancy of hydrogen interactions in the surfactin/*Pf*Sir2A complex compared to the NAD/*Pf*Sir2A complex highlights the importance of van der Waals interactions in stabilizing the surfactin binding. This information enhances our understanding of the molecular interactions underlying their potential inhibitory effects.

## Conclusion

In this work, we emphasize the significance of prospecting for bioactive molecules from endophytic microorganisms as a promising approach to discovering compounds with anti-plasmodium activity. Our study focused on the identification and characterization of six surfactins produced by endophytic bacteria from *P. splendens*, including the isoforms of the major compounds. Of particular interest, surfactin 2d-02 has been previously reported as a potent inhibitor of the Sir2A protein of *Plasmodium falciparum in vitro* studies. To further investigate their potential, we conducted extensive molecular dynamics simulations on surfactin 1d-02. Our results demonstrated that the *Pf*Sir2A 1d-02 complex exhibited more stable dynamics, as indicated by the RMSD and RMSF analyses, compared to the NAD complex. Moreover, surfactin 1d-02 displayed strong binding affinity as a *Pf*Sir2A inhibitor, with substantial binding energies calculated using both the MM/GBSA and SIE approaches. These findings underscore the structural compositions of surfactins as promising starting points for developing potential inhibitors against *P. falciparum*. The discovery of surfactins from endophytic bacteria provides valuable insights into harnessing these natural products as potential therapeutic agents against malaria disease.

## Data Availability

The raw data supporting the conclusion of this article will be made available by the authors, without undue reservation.
