# Supplementary material for: Molecular characterization and in silico evaluation of surfactins produced by endophytic bacteria from Phanera splendens
Source: Front Chem. 2023 Aug 7;11:1240704. doi: 10.3389/fchem.2023.1240704 (PMC10441774; doi:10.3389/fchem.2023.1240704)
Supplement: Supplementary file 1 [file DataSheet1.docx]

**SUPPORT INFORMATION**

**Molecular characterization and *in silico* evaluation of surfactins produced by Endophytic bacteria from *Phanera splendens***

Eleane Monaliza de Cerqueira de Souza^1^, Maycon Vinicius Damasceno de Oliveira^2^, José Edson de Sousa Siqueira^1^, Daniela Cristiane da Cruz Rocha^3^, **Anderson do Nonato Rosario Marinho**^3^**,** Andrey Moacir do Rosario Marinho^1^, Patrícia Santana Barbosa Marinho^1*^, Anderson H. Lima^2*^

^1^*Laboratório de Bioensaios e Química de Microrganismos, Instituto de Ciências Exatas e Naturais, Universidade Federal do Pará, 66075-110, Belém, Pará, Brasil.*

^2^*Laboratório de Planejamento e Desenvolvimento de Fármacos, Instituto de Ciências Exatas e Naturais, Universidade Federal do Pará, 66075-110, Belém, Pará, Brasil.*

^3^*Laboratório de Enteroinfecções Bacterianas, Instituto Evandro Chagas, 67030-000, Ananindeua, Pará, Brasil.*

^*^Corresponding author

E-mail: [anderson@ufpa.br](mailto:anderson@ufpa.br) (AHL) and [pat@ufpa.br](mailto:pat@ufpa.br) (PSBM)

Figure S1. The positive mode ESI mass spectra (*full scan*) for the surfactin derivatives group **1** (a-e).


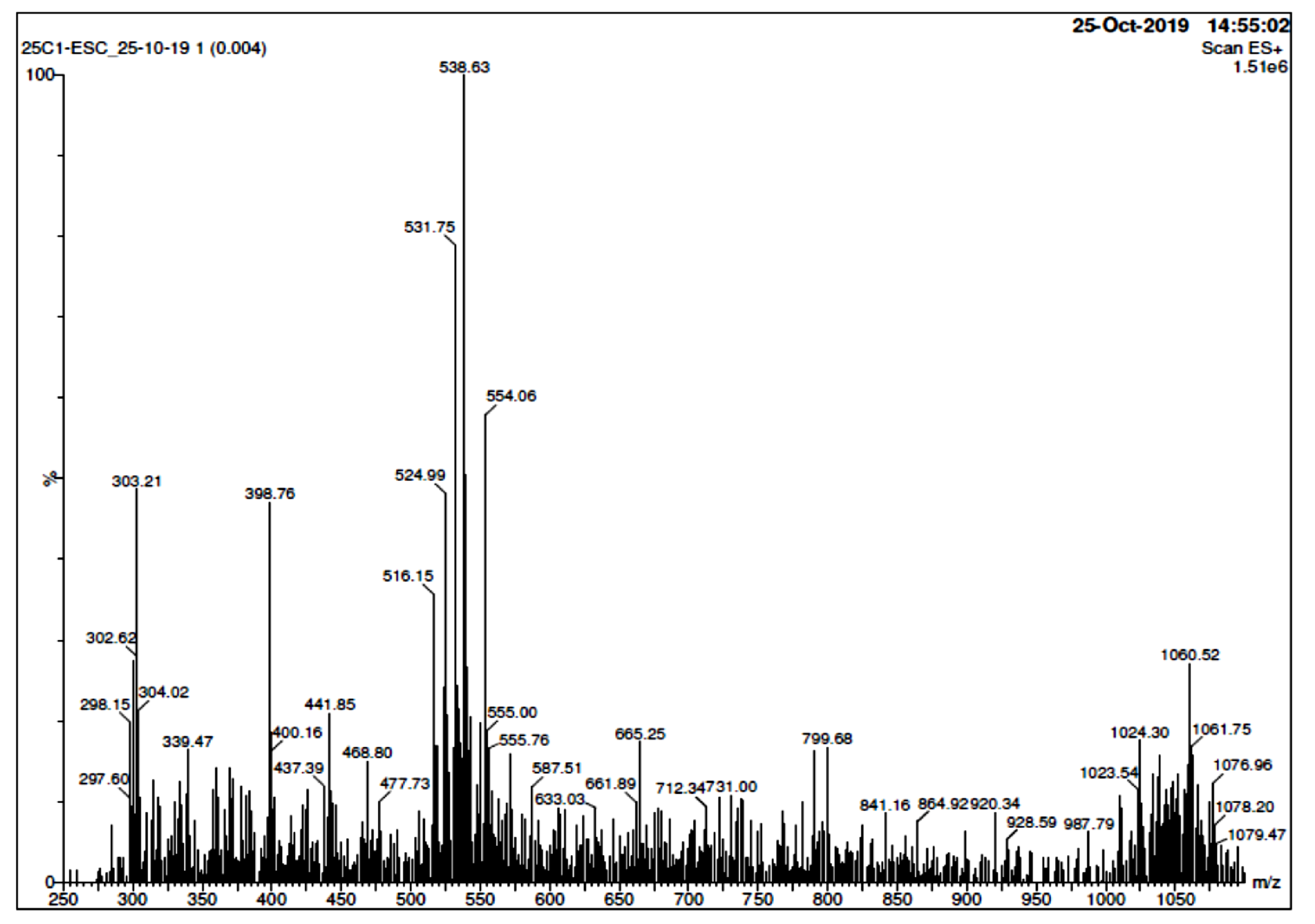


Figure S2. The negative mode ESI mass spectra (*full scan*) for the surfactin derivatives group **1** (a-e).
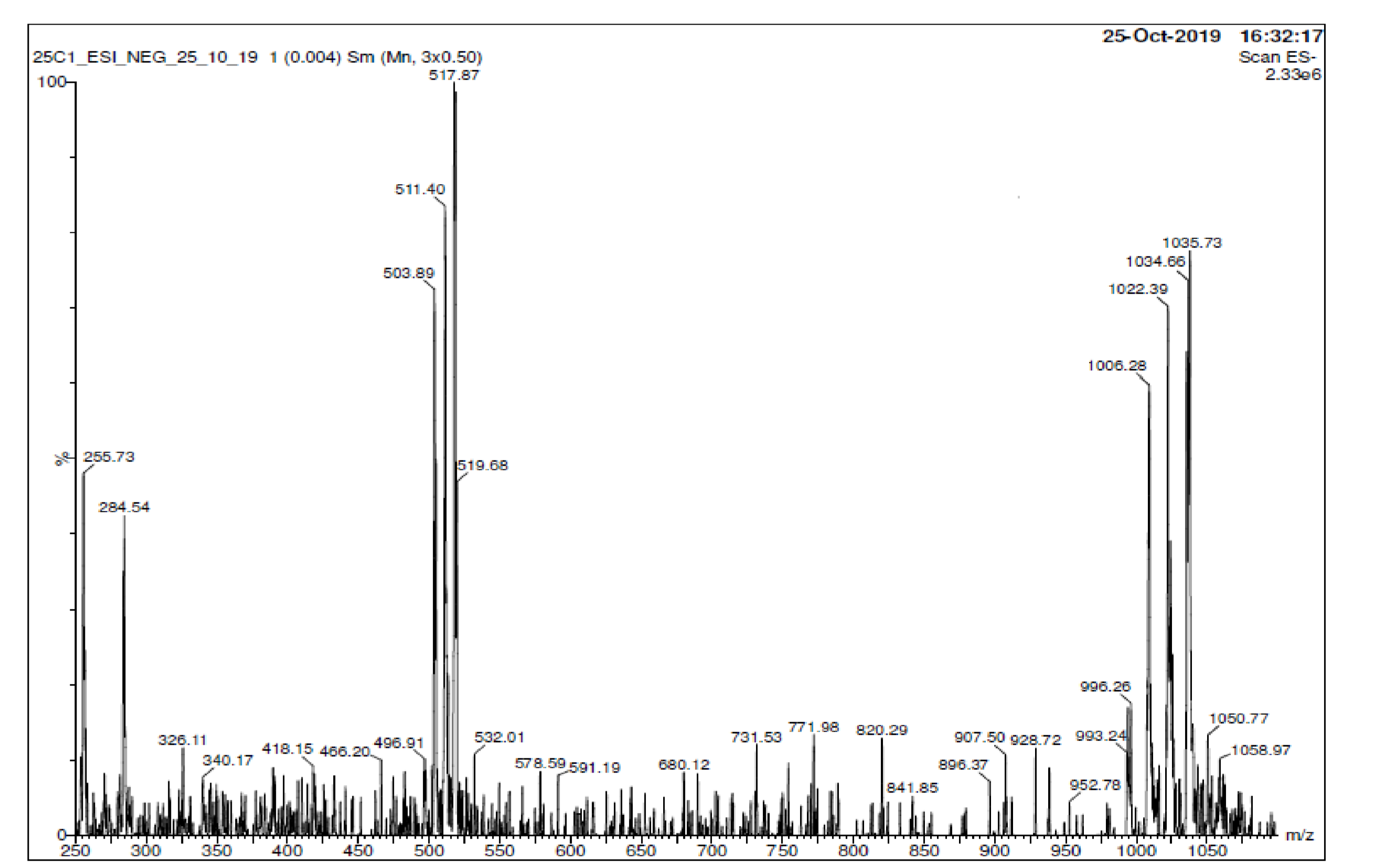


Figure S3. Mass spectrum of the daughter ions of the molecular ion *m/z* 1038 (**1d**).


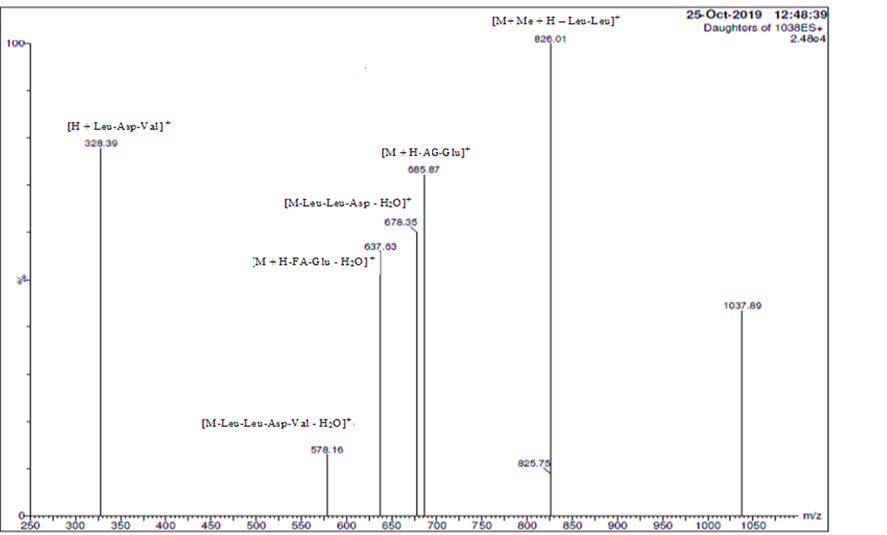
 Fonte: Elaborado pelo autor do trabalho.

Figure S4. The negative mode ESI mass spectra (*full scan*) for the surfactin derivatives group **2** (a-f).


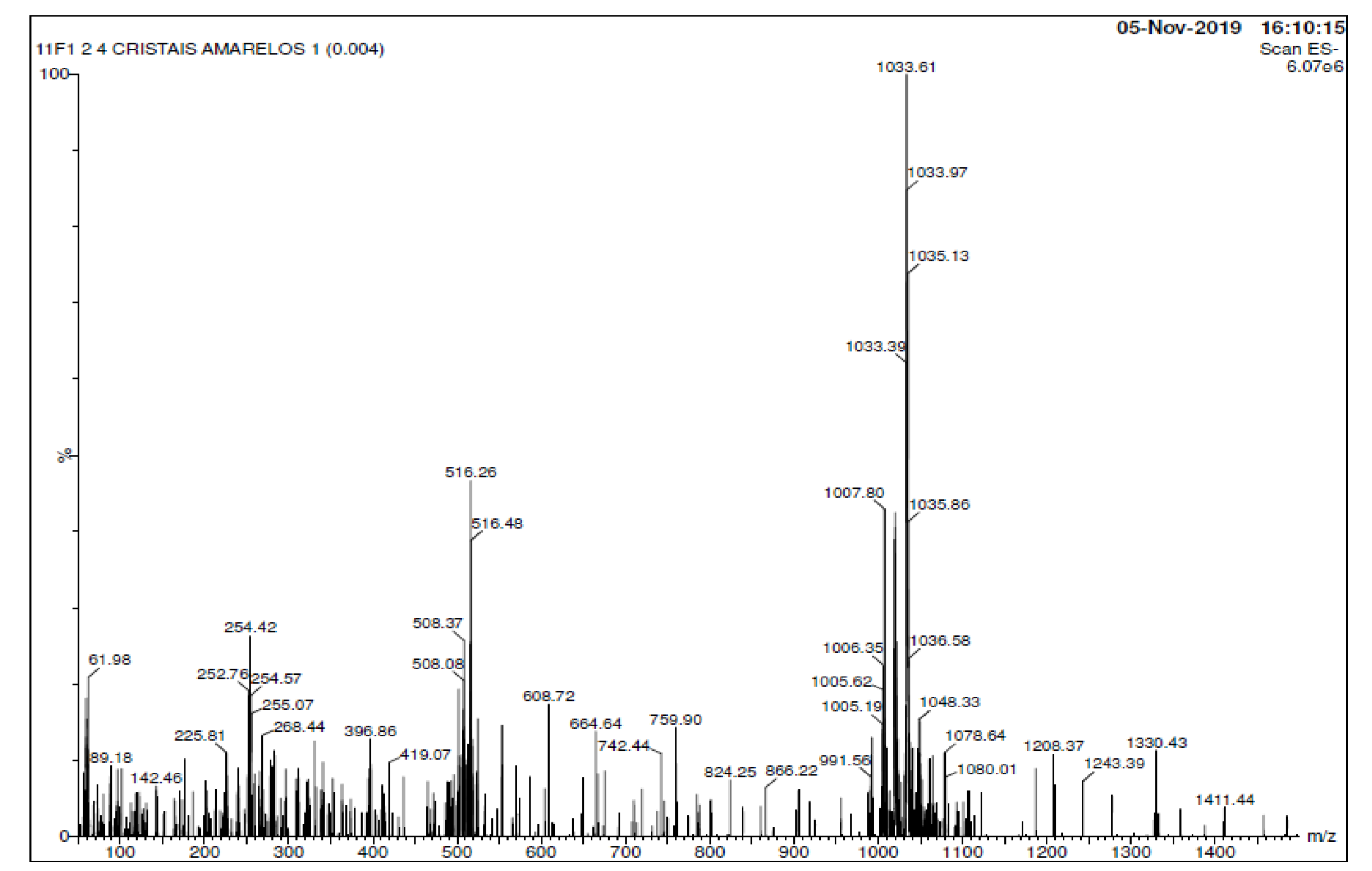


Figure S5. Mass spectrum of the daughter ions of the molecular ion *m/z* 1035 (**2d**).


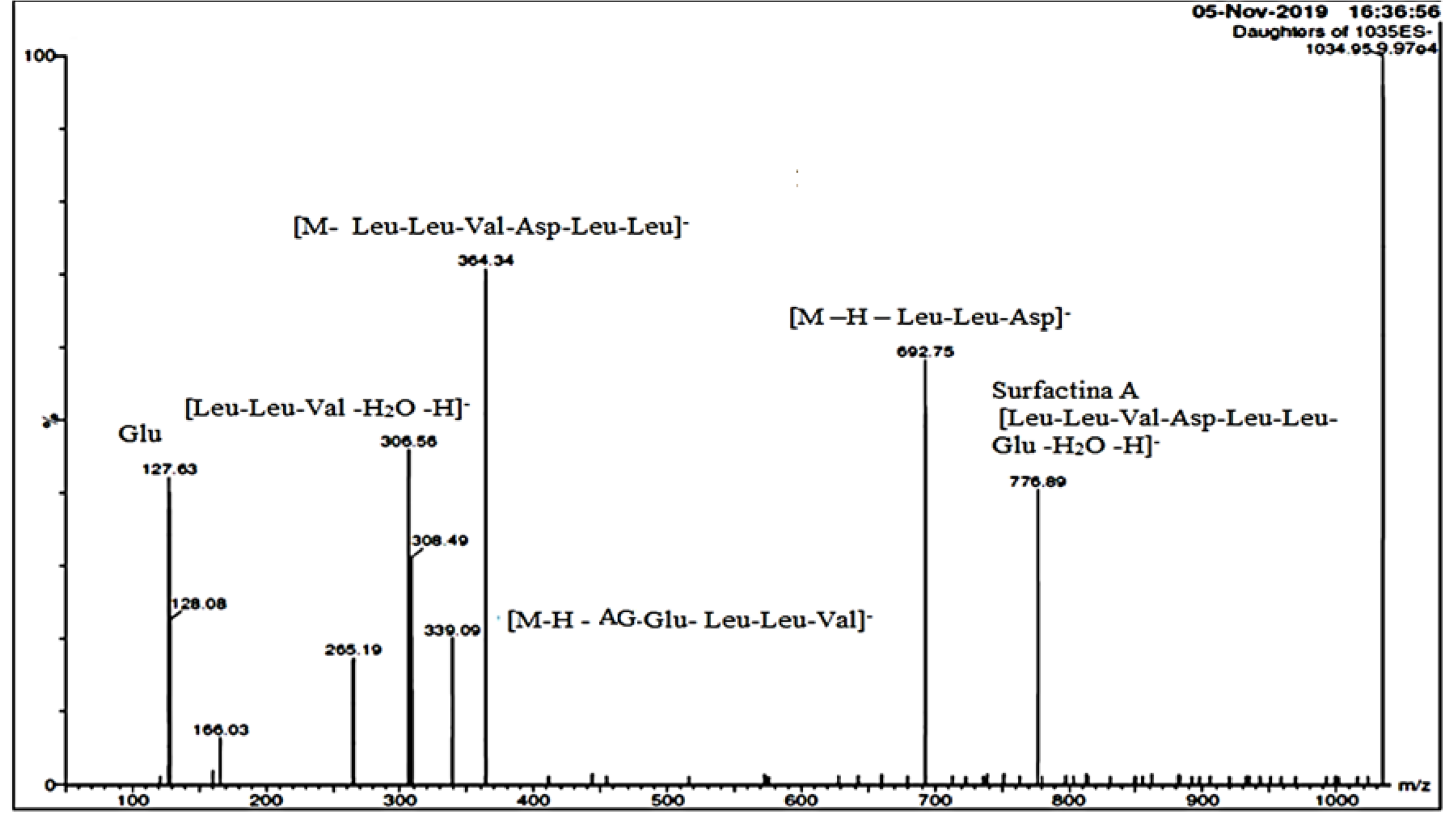
.

**Table S1:** Main hydrogen interactions obtained with the last 100 ns of the MD simulation with Surfactin/NAD Atom, Residue, Residue Atom, Average Distance Value in An and their respective Fraction of frames the bond percent.

| **Hydrogen Interactions** | | | | |
| --- | --- | --- | --- | --- |
| **Surfactin 1d-01** | | | | |
| **Surfactin**  **(Atom)** | **Residue** | **Residue**  **(Atom)** | **Average Distance**  **(Å)** | **Fraction**  **Frames** |
| O8 | Ser37 | OG | 2.67 | 38% |
| O6 | Trp56 | NE1 | 2.86 | 34% |
| O9 | Ser37 | OG | 2.68 | 26% |
| O1 | Thr211 | OG1 | 2.75 | 20% |
| O9 | Arg49 | N | 2.84 | 19% |
| O8 | Arg49 | N | 2.84 | 17% |
| O10 | Arg49 | NH1 | 2.82 | 15% |
| **Surfactin 1d-02** | | | | |
| **Surfactin (Atom)** | **Residue** | **Residue**  **(Atom)** | **Average Distance** | **Fraction Frames** |
| O12 | Tyr64 | OH | 2.76 | 35% |
| O4 | Arg49 | N | 2.87 | 32% |
| O7 | Arg49 | NH2 | 2.80 | 25% |
| O8 | Arg49 | NH2 | 2.80 | 23% |
| O8 | Ser37 | OG | 2.70 | 19% |
| O7 | Ser37 | OG | 2.71 | 16% |
| O7 | Arg49 | NH1 | 2.80 | 10% |
| **Surfactin 1d-03** | | | | |
| **Surfactin**  **(Atom)** | **Residue** | **Residue**  **(Atom)** | **Average**  **Distance** | **Fraction Frames** |
| O11 | Thr211 | OG1 | 2.77 | 55% |
| O9 | Arg49 | N | 2.84 | 30% |
| O8 | Arg49 | N | 2.84 | 28% |
| O8 | Gly50 | N | 2.85 | 21% |
| O9 | Gly50 | N | 2.85 | 19% |
| O11 | Ser209 | OG | 2.75 | 18% |
| **Surfactin 2d-01** | | | | |
| **Surfactin**  **(Atom)** | **Residue** | **Residue**  **(Atom)** | **Average**  **Distance** | **Fraction Frames** |
| O1 | Ser209 | OG | 2.68 | 29% |
| O | Ser209 | OG | 2.69 | 24% |
| 010 | Arg49 | N | 2.85 | 22% |
| O1 | Thr208 | OG1 | 2.70 | 21% |
| O1 | Ser209 | N | 2.85 | 19% |
| O | Thr208 | OG1 | 2.71 | 19% |
| O | Ser37 | OG | 2.68 | 19% |
| O | Ser209 | N | 2.85 | 14% |
| O1 | Thr211 | OG1 | 2.72 | 12% |
| O10 | Ser37 | OG | 2.71 | 12% |
| O1 | Ser37 | OG | 2.68 | 11% |
| O9 | Arg49 | NE | 2.84 | 10% |
| **Surfactin 2d-02** | | | | |
| **Surfactin**  **(Atom)** | **Residue** | **Residue**  **(Atom)** | **Average**  **Distance** | **Fraction Frames** |
| O12 | Arg49 | NH12 | 2.82 | 28% |
| O7 | Arg49 | NE | 2.85 | 19% |
| O6 | Arg49 | NE | 2.85 | 12% |
| O11 | Val116 | N | 2.87 | 11% |
| **Surfactin 2d-03** | | | | |
| **Surfactin**  **(Atom)** | **Residue** | **Residue**  **(Atom)** | **Average**  **Distance** | **Fraction Frames** |
| O | Ser213 | OG | 2.68 | 45% |
| O1 | Ser213 | OG | 2.68 | 35% |
| O1 | Ser213 | N | 2.86 | 31% |
| O | Ser213 | N | 2.86 | 28% |
| O8 | Ser52 | OG | 2.69 | 20% |
| O7 | Ser52 | OG | 2.69 | 18% |
| O8 | Ser52 | N | 2.86 | 14% |
| O7 | Ser52 | N | 2.86 | 13% |
| **NAD** | | | | |
| **NAD**  **(Atom)** | **Residue** | **Residue**  **(Atom)** | **Average**  **Distance** | **Fraction Frames** |
| O8 | Ser209 | OG | 2.66 | 89% |
| O9 | Thr208 | OG1 | 2.75 | 80% |
| H14 | Asn233 | OD1 | 2.69 | 71% |
| O8 | Ser209 | N | 2.85 | 65% |
| O12 | Asn233 | ND2 | 2.87 | 64% |
| N5 | Phe252 | N | 2.90 | 56% |
| O9 | Ser37 | N | 2.87 | 40% |
| O8 | Thr208 | HG1 | 2.76 | 17% |
